# A role for MCP-1/CCR2 in interstitial lung disease in children

**DOI:** 10.1186/1465-9921-6-93

**Published:** 2005-08-11

**Authors:** Dominik Hartl, Matthias Griese, Thomas Nicolai, Gernot Zissel, Christine Prell, Dietrich Reinhardt, Dolores J Schendel, Susanne Krauss-Etschmann

**Affiliations:** 1 Childrens' Hospital of the Ludwig-Maximilians-University, Munich, Germany; 2 Department of Pneumology, Medical Center, Albert-Ludwigs-University, Freiburg, Germany; 3 Institute of Molecular Immunology and Immune Monitoring Platform, GSF National Research Center for Environment and Health, Munich, Germany

**Keywords:** Chemokines, MCP-1, CCR2, Bronchoalveolar Lavage, Children, Interstitial Lung Diseases

## Abstract

**Background:**

Interstitial lung diseases (ILD) are chronic inflammatory disorders leading to pulmonary fibrosis. Monocyte chemotactic protein 1 (MCP-1) promotes collagen synthesis and deletion of the MCP-1 receptor CCR2 protects from pulmonary fibrosis in ILD mouse models. We hypothesized that pulmonary MCP-1 and CCR2^+ ^T cells accumulate in pediatric ILD and are related to disease severity.

**Methods:**

Bronchoalveolar lavage fluid was obtained from 25 children with ILD and 10 healthy children. Levels of pulmonary MCP-1 and Th1/Th2-associated cytokines were quantified at the protein and the mRNA levels. Pulmonary CCR2^+^, CCR4^+^, CCR3^+^, CCR5^+ ^and CXCR3^+ ^T cells were quantified by flow-cytometry.

**Results:**

CCR2^+ ^T cells and MCP-1 levels were significantly elevated in children with ILD and correlated with forced vital capacity, total lung capacity and ILD disease severity scores. Children with lung fibrosis had significantly higher MCP-1 levels and CCR2^+ ^T cells in bronchoalveolar lavage fluid compared to non-fibrotic children.

**Conclusion:**

The results indicate that pulmonary CCR2^+ ^T cells and MCP-1 contribute to the pathogenesis of pediatric ILD and might provide a novel target for therapeutic strategies.

## Background

Interstitial lung diseases (ILD) are chronic inflammatory disorders characterized by restrictive lung disease and diffuse pulmonary infiltrates. Although the precise incidence is not known, ILD are less frequent in children than adults [1-3]. Lungs of ILD patients show inflammation with alveolar wall thickening by leukocytes and pulmonary fibrosis. Despite immunosuppressive treatment and supportive measures, the progressive course leading to irreversible lung fibrosis sometimes can not be prevented. Therefore, the development of additional therapeutic strategies is of high importance.

Monocyte chemotactic protein 1 (MCP-1, CCL2) is produced in response to inflammatory stimuli by a variety of cells, including monocytes/macrophages, lymphocytes and airway epithelial cells [4-6]. MCP-1 stimulates collagen synthesis and production of the pro-fibrotic factor transforming growth factor β (TGF-β) in fibroblasts, while MCP-1 antisense oligonucleotides reduce TGF-β production[7,8]. Application of MCP-1 into murine lungs induces an inflammatory cytokine response and pulmonary leukocyte accumulation. In adult patients with ILD, increased levels of MCP-1 were observed in serum[9,10] and bronchoalveolar lavage fluid (BALF) [11-14]. Although MCP-1 was originally described for its chemotactic activity on monocytes, *in vitro *studies revealed an even higher activity on T cells[15]. This occurs through MCP-1 binding to its sole receptor CCR2[16]. Deletion of the CCR2-gene or receptor blockade with anti-CCR2 antibodies leads to a dramatic inhibition of leukocyte accumulation in murine lungs[17]. Furthermore, CCR2-/- mice are protected from fluorescein (FITC) or bleomycin induced lung fibrosis[18]. Thus far, CCR2^+ ^T cells in BALF of patients with fibrotic lung diseases have not been determined.

In addition to the MCP-1/CCR2 axis, Th2 cytokines seem to mediate pulmonary fibrosis [19-22]. IL-4 stimulates fibroblast proliferation and collagen synthesis[23,24], while IFN-γ inhibits this process [25-28]. In a Th2 mouse model fibroblasts expressed more CCR2 protein and higher levels of MCP-1 and TGF-β as compared to fibroblasts from a Th1-mouse model[8]. Furthermore, increased levels of IL-4 were observed in animal models of pulmonary fibrosis[29] and lungs of patients with idiopathic pulmonary fibrosis (IPF)[30] or cryptogenic fibrosing alveolitis[31].

The contribution of MCP-1 to ILD has been investigated exclusively in adults. However, the spectrum of ILD differs considerably between adults and children and some forms are unique to children while others, such as idiopathic pulmonary fibrosis (IPF), are extremely rare in childhood[32].

Therefore, we asked whether levels of MCP-1 and frequencies of CCR2^+ ^T cells are increased in BALF of children with ILD and, if so, how levels of MCP-1 and CCR2^+ ^T cells relate to disease severity in pediatric ILD.

To address these questions levels of MCP-1 and frequencies of CCR2^+ ^T cells in BALF were compared between children with ILD and children without lung disease.

To evaluate the contribution of the pulmonary Th1/Th2 micromilieu to the pathogenesis of pediatric ILD, CCR4^+ ^and CCR3^+ ^(Th2) and CCR5^+ ^and CXCR3^+ ^(Th1) cells were determined in BALF together with an array of pulmonary Th1- and Th2-associated cytokines.

Our results indicate that pulmonary CCR2^+ ^T cells and levels of MCP-1 are characteristic components in BALF of children with ILD. A pathophysiological role in pediatric ILD seems likely as their levels relate to restrictive lung function and ILD disease severity.

## Methods

### Characterization of the patients

Children attending the Department of Pulmonology and Allergology of the University Children's Hospital of Munich during 1999–2004 were considered for inclusion in this study. Children suspective of ILD underwent a comprehensive clinical evaluation, including patient history, physical examination, routine laboratory tests, lung function testing, chest radiography, high resolution computed tomography (HRCT) and bronchoalveolar lavage (BAL). Children were assigned to the ILD group according to the criteria of Fan[33]: (i) ≥3 months of respiratory symptoms characteristic for ILD, i.e. non-productive cough, dyspnoea, tachypnea, crackles and/or rales, exercise intolerance and/or hypoxemia, (ii) diffuse infiltrates on chest radiographs and HRCT and (iii) restrictive lung function (decreased forced vital capacity (FVC) and total lung capacity (TLC)) according to the ATS criteria[34].

The diagnosis of the specific form of ILD was established by patient history, physical examination, HRCT, BAL and/or lung biopsy according to consensus criteria[33,35]. Two thoracic radiologists independently evaluated all lobes on HRCT for ground glass opacity and pulmonary fibrosis as described previously[36,37]. A pathologist specialized on pediatric ILD[38] evaluated the lung sections systematically[39,40]. Furthermore, the disease severity of each ILD patient was characterized using the clinical ILD score of Fan[41]: 1 = asymptomatic, no desaturation; 2 = symptomatic but normoxic (>90%) under all conditions; 3 = symptomatic with desaturation during sleep or with exercise; 4 = symptomatic with desaturation at rest. None of the included children had familial idiopathic pulmonary fibrosis. Patients with congenital heart disease or suspected or proven bacterial pulmonary infection were excluded from the study.

Twenty-five children with ILD (median age: 7 ± 3.6 years; male/female = 16/9) were included (Table 1).

**Table 1:** Patients' characteristics

**No**	**Sex**	**Age **[years]	**Interstitial lung disease**	**Diagnosis finding**	**Radiographic findings**	**Fibrotic changes (CT)**	ILD Score*	**Dyspnoe**	**Cough**	**Cyanosis**	**Exercise Intolerance**	**Failure to thrive**	**Medication**	**FVC [% of pred.]**	**TLC % [of pred.]**
1	F	7	LIP	CT, LB	• diffuse interstitial involvement	+	4	++	+	+	+	+	CS, AZT	34	56
					• reticular-nodular pattern										
					• follicular bronchiolitis										
2	M	14	U-ILD, IPH	CT, BAL	patchy interstitial involvement	-	2	+	-	-	-	-	CS	77	89
3	M	8	U-ILD	CT, LB	• ground-glass opacity	+	3	++	-	-	+	-		46	74
4	M	4	IPH	CT, BAL, LB	interstitial involvement	-	3	+	-	-	-	-		77	168
5	M	16	U-ILD	CT, BAL	interstitial involvement	+	2	+	-	-	+	+		76	95
6	F	7	U-ILD	CT, BAL	interstitial involvement	-	2	+	+	-	-	+	AZT	50	68
7	M	4	CPI	CT, LB	• diffuse infiltrates	+	3	+	-	-	+	+	AZT	58	64
					• ground-glass opacity										
8	F	3	NSIP	CT, LB	• interstitial involvement	+	3	++	-	+	+	+	CS	n.d.	n.d.
					• alveolar infiltrates										
9	M	8	Sarcoidosis	CT, BAL, LB	• interstitial involvement	+	2	++	+	-	+	+	CS	56	63
					• perivascular nodules										
10	F	8	U-ILD	CT, BAL, LB	ground-glass opacity	-	1	-	+	-	+	-		76	87
11	F	8	CPI	CT, LB	• interstitial involvement	+	2	+	+	-	+	+	CS	37	74
					• ground-glass opacity										
12	M	9	U-ILD	CT	interstitial involvement	-	2	+	-	-	-	-		70	98
13	M	5	NSIP	CT, LB	• interstitial involvement	+	3	++	-	-	+	-	CS	61	76
					• ground-glass opacity										
14	F	6	U-ILD	CT	reticular-nodular pattern	+	3	++	+	-	-	-	AZT	60	68
15	F	4	U-ILD	CT	interstitial involvement	+	2	+	+	-	-	+		n.d.	n.d.
16	M	12	U-ILD	CT	interstitial involvement	-	2	+	-	-	-	-		68	75
17	M	3	PAP†	CT, BAL, LB	• ground glass opacity	-	4	+++	+	+	+	+	CS	n.d.	n.d.
18	M	6	NSIP	CT, BAL, LB	• alveolar infiltrates	+	4	+++	+	+	+	+	CS, AZT	63	72
			PAP		• ground glass opacification										
19	F	3	PAP†	CT, BAL, LB	• ground glass opacity	+	4	++	-	+	+	+	CS	n.d.	n.d.
					• alveolar infiltrates										
20	F	9	NSIP	CT, LB	• interstitial involvement	+	3	++	+	+	+	+	CS, AZT	55	74
					• honeycombing										
21	M	7	U-ILD	CT	reticular-interstitial pattern	+	3	+	+	-	+	+	AZT, MT	38	59
22	M	7	Cholesterol	CT, BAL, LB	• interstitial involvement	+	4	+++	+	+	+	+	CS	16	24
			pneumonitis†		• reticular-interstitial pattern										
23	M	4	U-ILD	CT, LB	• interstitial involvement	-	2	+	-	-	+	+	CS	102	99
					• honeycombing										
24	M	8	U-ILD	CT	interstitial involvement	-	2	+	+	-	+	-	CS	63	78
25	M	7	NSIP	CT, LB	• interstitial involvement	+	3	+	+	-	+	-	CS	60	76

ILD-NC: children with interstitial lung disease without systemic corticosteroid treatment; ILD-C: children with interstitial lung disease with systemic corticosteroid treatment;

U-ILD: undefined/idiopathic interstitial lung disease: no specific diagnosis could be made; PAP: pulmonary alveolar proteinosis; CGD: chronic granulomatous disease; IPH: idiopathic pulmonary hemosiderosis; LIP: lymphocytic interstitial pneumonia; CPI: Chronic pneumonitis of infancy

CS: corticosteroids, AZT: azathioprine, MT: methotrexat

n.d.: lung function testing not done (children < 5 years); † symbolizes patients who died due to respiratory failure.

CT: Computed tomography; BAL: Bronchoalveolar lavage; LB: Lung biopsy

* ILD score according to Fan[41]

Ten age-matched children were selected as the control group (median age: 7.5 ± 2.9 years, m/f: 6/4). These children were considered as healthy, i.e. had no systemic disease, had no suspected or proven pulmonary disease and were free of respiratory tract infections. These children underwent elective tonsillectomy under general anaesthesia. BAL was performed prior to the surgical procedure.

Ten age-matched children with chronic severe asthma (median age: 8.7 ± 1.6 years, m/f: 5/5), from a previous study[42], who were comparable to the ILD group in terms of gender and age were included as disease control group. All parents and/or patients gave their informed consent prior to bronchoscopy and the institutional review board approved the study protocol.

### Bronchoalveolar lavage

Bronchoscopy with BAL was performed as described previously[43]. Residual BALF cells were used for flow cytometry. The BALF recovery and the viability of cells did not differ significantly between the patient groups. Cellular profiles are shown in Table 2.

**Table 2:** Bronchoalveolar lavage cells

	ILD-NC	ILD-C	Control
Total cells × 10^3^/ml	230 (2.1–1124)**	144 (11–268)*	89 (83–97)
Recovery (%)	55 (25–86)	49 (34–75)	54 (35–70)
Neutrophils (%)	10.5 (1–44)*	8.5 (3–30)*	2 (0–3)
Eosinophils (%)	1 (0–6)	1.5 (0–3)	0 (0–1)
Mast cells (%)	2 (0–43)	2 (1–4)	0 (0-0)
Plasma cells (%)	0 (0–4)	0 (0–4)	0 (0-0)
Macrophages (%)	60 (7–97)*	49 (26–77)*	94 (81–92)
Lymphocytes (%)	24 (2–54)**	22 (5–34)**	4 (2–13)
CD4^+ ^T cells (%)^†^	23 (9–45)	29 (9–82)	23 (15–28)
CD8^+ ^T cells (%)^†^	29 (6–62)	27 (2–83)	25 (15–31)
CD4/8 ratio	0.7 (0.3–6)	1.1 (0.1–55)	0.7 (0.4–0.9)

results are expressed as medians with ranges shown in parenthesis.

ILD-NC: children with interstitial lung disease without systemic corticosteroid treatment;

ILD-C: children with interstitial lung disease with systemic corticosteroid treatment;

*p < 0.05, **p < 0.01 as compared to the control group, Mann-Whitney-*U *Test.

Total cells and differential cell count were obtained from cytospin slides, CD4^+^, CD8^+ ^and CD4/CD8 T cells using flow cytometry.

^†^CD4^+ ^T cells and CD8^+ ^T cells are shown as the percentage of total lymphocytes in BALF, i.e. cells gated in the lymphocyte population. Neutrophils, eosinophils, mast cells, plasma cells, macrophages and lymphocytes are shown as percentage of total cells in BALF.

### Flow cytometry

BALF cells were analyzed by four-colour flow cytometry (FACSCalibur, Becton-Dickinson, Heidelberg, Germany) as described previously[42]. The following antibodies were used: CD4-allophycocyanine (APC) mouse IgG1, CD8-phycocyanine 5 (PC5) mouse IgG1 (Immunotech, Marseille, France), CD69-PE mouse IgG1, CCR5-PE mouse IgG2a, CCR4-PE mouse IgG2a (BD Pharmingen, Heidelberg, Germany), CCR2-PE mouse IgG2b, CXCR3-fluorescein isothiocyanate (FITC) mouse IgG1 and CCR3-FITC rat IgG2a (R&D Systems, Wiesbaden, Germany). Mouse IgG1-FITC, mouse IgG1-PE, mouse IgG2a-PE, mouse IgG2b-PE (Immunotech, Marseille, France) and rat IgG2a-FITC (kindly provided by Dr. E. Kremmer, GSF-Institute of Molecular Immunology, Munich, Germany) were used as isotype controls.

### Detection of MCP-1 and cytokines

Levels of MCP-1 and Th1 (IL-2, IFN-γ), Th2 (IL-4, IL-5, IL-10) and pro-inflammatory cytokines (TNF-α, IL-6) were quantified by a multiplex, particle-based assay (Bio-Rad Laboratories, Minneapolis, USA) as described previously[42]. The detection limits for all cytokines were 1.5–2.5 pg/ml (min.) and 1000 pg/ml (max.).

### Quantitative RT-PCR

BALF cells were lysed in Trizol LS Reagent (Invitrogen, Life Technologies, Karlsruhe, Germany) and were stored at -20°C until mRNA extraction. Total mRNA was isolated according to the manufacturer's instructions and reverse transcribed into cDNA. Contamination with genomic DNA was excluded by mRNA controls without reverse transcriptase in the cDNA synthesis reaction. The following oligonucleotide primers were used: MCP-1 (5-TGAAGCTCGCACTCTCGCCT-3; 5- GTGGAGTGAGTGTTCAAGTC-3); and GAPDH (5-GAGGTGAAGGTCGGAGTC-3; 5-AAGATGGTGATGGGATTTC-3). Expression levels were determined in duplicates by Real time RT-PCR using SYBR green and the iCycler iQ detection system (Biorad, Hercules, CA, USA) according to the manufacturer's instructions. Threshold cycle (CT) values for genes of interest were normalized to GAPDH and used to calculate the relative mRNA expression.

### Statistical analysis

The non-parametric Mann-Whitney *U *test was applied. Correlations were tested with Spearman's rank correlation test. A probability of p < 0.05 was regarded as significant[44] (SPSS statistical program, version 11.5, SPSS Inc. Chicago, USA).

## Results

### MCP-1 levels and CCR2^+ ^T cells in BALF

**Figure 1:**
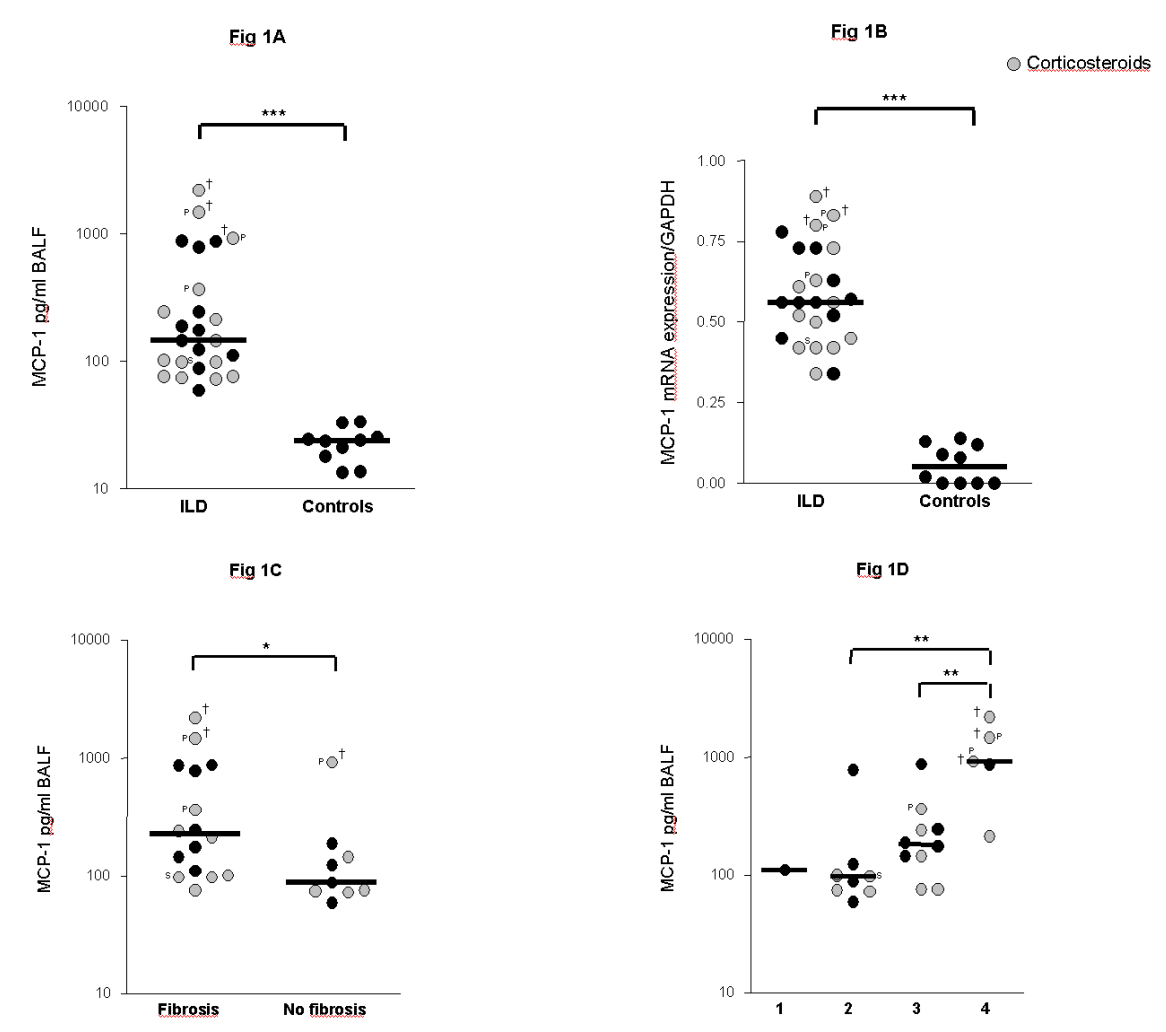
**MCP-1 levels in children with ILD**. MCP-1 levels in bronchoalveolar lavage fluid (BALF) of children with interstitial lung diseases (ILD) and healthy controls are shown at the (A) protein and at the (B) mRNA level. (C) MCP-1 levels in BALF of ILD children with and without pulmonary fibrosis. Pulmonary fibrosis was assessed by computed tomography according to [36,37]. (D) MCP-1 levels in ILD children related to ILD disease severity according to the criteria of Fan [33]. 1 = asymptomatic, no desaturation; 2 = symptomatic but normoxic (> 90%) under all conditions; 3 = symptomatic with desaturation during sleep or exercise; 4 = symptomatic with desaturation at rest; MCP-1 protein levels were quantified in BALF by a multiplex, particle-based assay (Bio-Rad Laboratories, Minneapolis, USA) as described previously [42]. MCP-1 mRNA levels were quantified in BALF cells by Real time RT-PCR using SYBR green and the iCycler iQ detection system (Biorad, Hercules, CA, USA) and were normalized to GAPDH. Median values are shown by horizontal bars. Differences between the patient groups were tested with the Mann-Whitney *U *test; * p < 0.05, *** p < 0.001; Children with systemic corticosteroid therapy are shown as grey circles. P: Pulmonary alveolar proteinosis; S: Sarcoidosis; † symbolize children who died due to respiratory failure.

**Figure 2:**
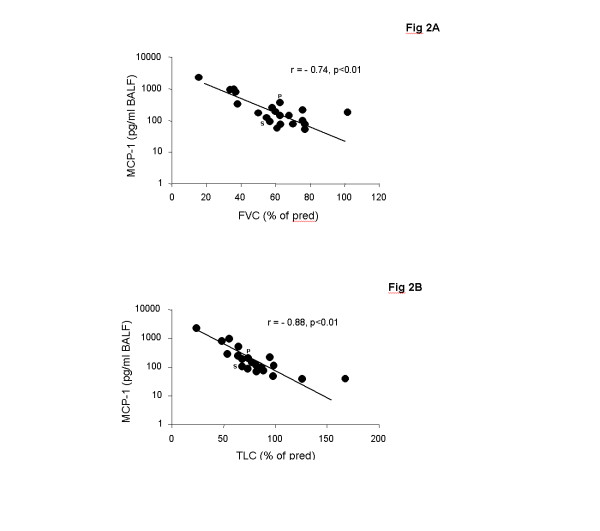
**Correlation of MCP-1 levels with lung function parameters in children with ILD**. MCP-1 levels in bronchoalveolar lavage fluid (BALF) correlated with (A) forced vital capacity (FVC) and (B) total lung capacity (TLC) in children with interstitial lung disease (ILD). FVC and TLC are shown as % of predicted. MCP-1 levels in BALF were quantified by a multiplex, particle-based assay; P: Pulmonary alveolar proteinosis; S: Sarcoidosis;

Levels of MCP-1 were significantly higher in children with ILD (n = 25) as compared to the control group at protein and mRNA level (Figure 1A, B). MCP-1 protein and mRNA expression levels correlated positively with each other (r = 0.72, p < 0.01). ILD children with pulmonary fibrosis had significantly higher MCP-1 levels in BALF as compared to children with non-fibrotic ILD (Figure 1C). MCP-1 levels related positively to the stage of disease (Figure 1D). The highest levels of MCP-1 were observed in the three patients who died after respiratory failure (Table 1; P17, P19, P22). Furthermore, MCP-1 levels correlated negatively with restrictive lung function parameters (TLC, FVC) (Figures 2A, B).

**Figure 3:**
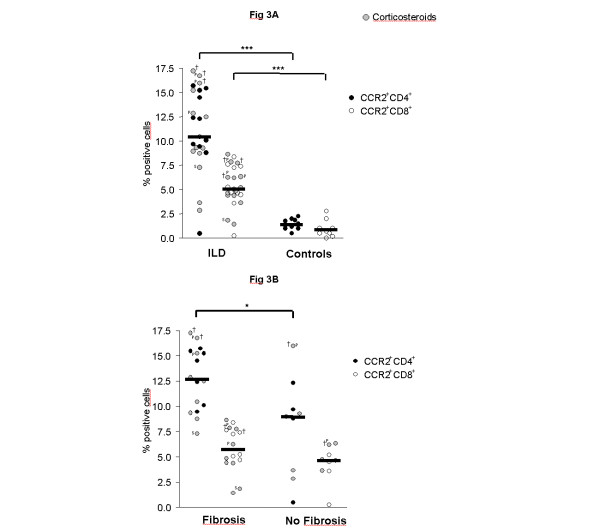
**CCR2^+ ^T cells in children with ILD**. (A) Percentages of CCR2^+^CD4^+ ^and CCR2^+^CD8^+ ^T cells in in bronchoalveolar lavage fluid (BALF) of children with interstitial lung diseases (ILD) and healthy children. (B) Percentages of CCR2^+^CD4^+ ^and CCR2^+^CD8^+ ^T cells in BALF of children with and without pulmonary fibrosis. Percentages of CCR2^+^CD4^+ ^and CCR2^+^CD8^+ ^T cells were analyzed in BALF by flow cytometry. Pulmonary fibrosis was assessed by computed tomography according to [36,37]. Median values are shown by horizontal bars. Differences between the patient groups were tested with the Mann-Whitney *U *test; * p < 0.05; *** p < 0.001; Children with systemic corticosteroid therapy are shown as grey circles. P: Pulmonary alveolar proteinosis; S: Sarcoidosis; † symbolize the children who died due to respiratory failure.

**Figure 4:**
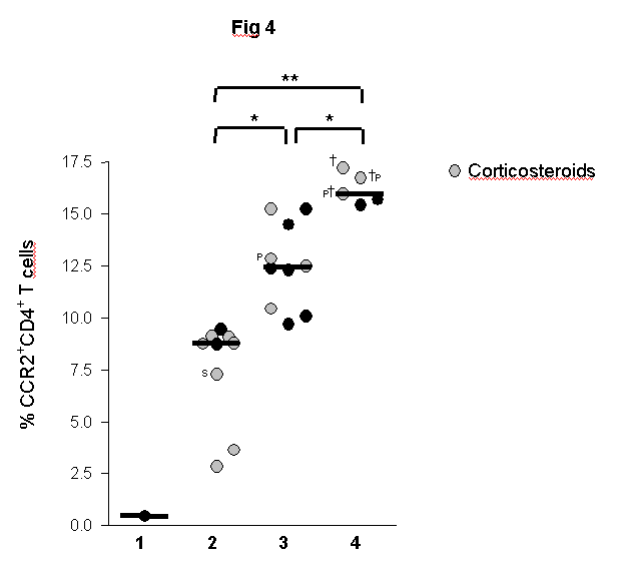
**CCR2^+ ^CD4^+ ^T cells and ILD disease severity**. Percentages of CCR2^+^CD4^+ ^T cells in bronchoalveolar lavage fluid (BALF) of children with interstitial lung disease (ILD) related to ILD disease severity. Percentages of CCR2^+^CD4^+ ^T cells were analyzed in BALF by flow cytometry. ILD disease severity was scored according to the ILD score of Fan(40): 1 = asymptomatic, no desaturation; 2 = symptomatic but normoxic (> 90%) under all conditions; 3 = symptomatic with desaturation during sleep or with exercise; 4 = symptomatic with desaturation at rest; Median values are shown by horizontal bars. Differences between the patient groups were tested with the Mann-Whitney *U *test; * p < 0.05, ** p < 0.01; Children with systemic corticosteroid therapy are shown as grey circles. P: Pulmonary alveolar proteinosis; S: Sarcoidosis; † symbolize the children who died due to respiratory failure.

**Figure 5:**
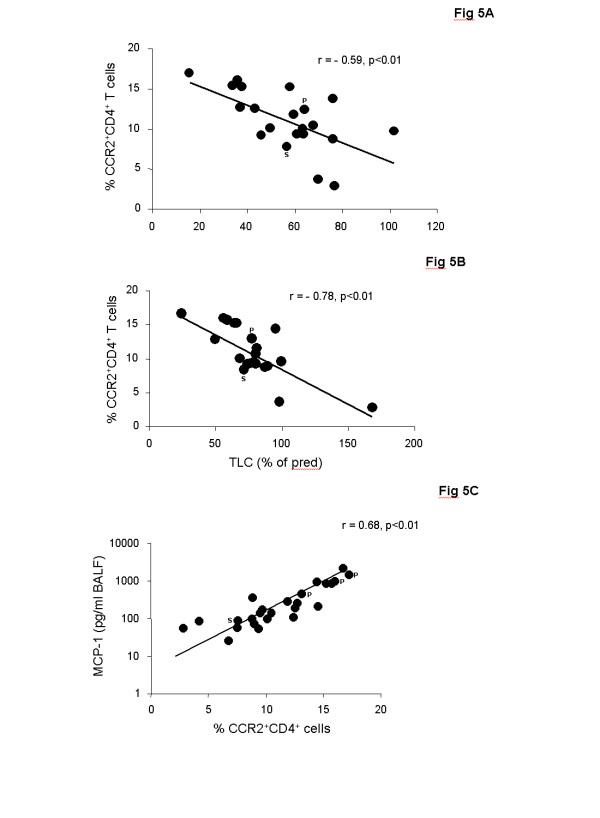
**CCR2^+^CD4^+ ^T cells and lung function parameters in children with ILD**. Correlation of CCR2^+^CD4^+ ^T cells in bronchoalveolar lavage fluid (BALF) with (A) forced vital capacity (FVC) and (B) total lung capacity (TLC) in children with interstitial lung diseases (ILD). Correlation of percentages of CCR2^+^CD4^+ ^T cells with levels of MCP-1 in BALF of children with ILD (C). FVC and TLC are shown as % of predicted. Percentages of CCR2^+^CD4^+ ^T cells were analyzed in BALF by flow cytometry. P: Pulmonary alveolar proteinosis; S: Sarcoidosis

To test whether increased MCP-1 levels are associated with increased frequencies of CCR2^+ ^T cells, BALF lymphocytes were quantified by flow cytometry. CCR2 was expressed on a higher percentage of CD4^+ ^than CD8^+ ^T cells. The majority of CCR2^+ ^T cells showed an activated phenotype (75% CCR2^+^CD69^+^). Children with ILD had significantly higher percentages of CCR2^+^CD4^+^and CCR2^+^CD8^+ ^T cells in BALF as compared to control children (Figure 3A). Similar to MCP-1, percentages of CCR2^+^CD4^+ ^cells were significantly higher in ILD children with pulmonary fibrosis as compared to children with non-fibrotic ILD (Figure 3B). Again, the highest percentages of CCR2^+^CD4^+ ^T cells were observed in the three deceased patients and CCR2^+^CD4^+ ^cells related positively to the stage of ILD (Figure 4). Furthermore, percentages of CCR2^+^CD4^+ ^T cells correlated negatively with FVC and TLC in ILD patients (Figures 5A, B). Pulmonary levels of MCP-1 correlated positively with CCR2^+^CD4^+ ^T cells (Figure 5C). No association between MCP-1/CCR2^+ ^cells and immunosuppressive treatment was found in ILD patients.

To verify if increased levels of MCP-1 and percentages of CCR2^+ ^T cells are characteristic for pediatric ILD, we analyzed these markers in ten selected age-matched children with well-characterized allergic asthma who are described in detail in a previous study[42]. Levels of MCP-1 and CCR2^+ ^T cells from asthmatic children were in the range of the control group and did not correlate with each other (data not shown).

**Figure 6:**
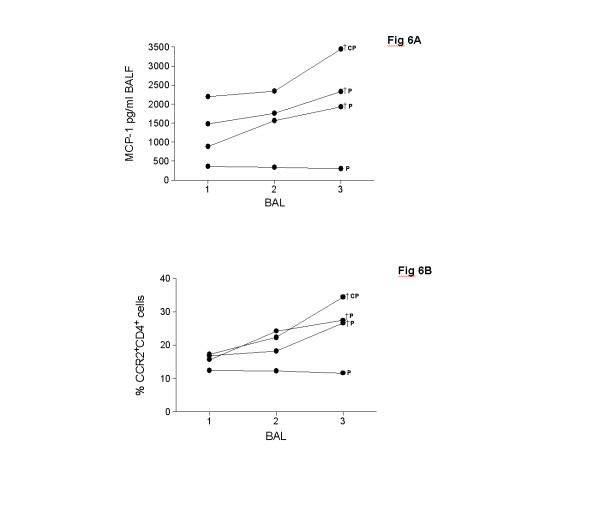
**Longitudinal analysis of MCP-1 levels and CCR2^+^CD4^+ ^T cells**. Longitudinal analysis of (A) MCP-1 levels and (B) CCR2^+^CD4^+ ^T cells in three consecutive bronchoalveolar lavage fluids (BALF) of four children with interstitial lung diseases, including two children with pulmonary alveolar proteinosis (P) and one child with cholesterol pneumonitis (CP). The child with cholesterol pneumonitis and one child with pulmonary alveolar proteinosis died by respiratory failure (†), while one child with pulmonary alveolar proteinosis stayed clinically stable. † symbolize the childen who died. MCP-1 levels were quantified in BALF by a multiplex, particle-based assay. Percentages of CCR2^+^CD4^+ ^T cells were analyzed in BALF by flow cytometry.

To assess the value of CCR2^+^CD4^+ ^T cells and MCP-1 levels in the longitudinal course, three consecutive therapeutical BALs were analyzed in three patients with PAP (P17, P18, P19) and one patient with cholesterol pneumonitis (P22). Two PAP patients (P17, P19) and the patient with cholesterol pneumonitis worsened in the clinical course continuously (increasing oxygen requirement, increasing dyspnoe) and died from respiratory failure, while one PAP patient remained clinically stable (P18). The deceased PAP patients had continuously rising levels of MCP-1 and increasing percentages of CCR2^+^CD4^+ ^T cells in the three follow-up BALs (Figures 6A, B; black circles) while the clinically stable patient showed steady levels of MCP-1 and percentages of CCR2^+^CD4^+ ^T cells (Figures 6A, B; white circles).

### Th1- and Th2-lymphocytes and cytokines in BALF

To test whether increased CCR2^+ ^T cells and levels of MCP-1 were paralleled by a pulmonary Th1/Th2-shift, CCR4^+ ^and CCR3^+^(Th2) and CCR5^+^an d CXCR3^+ ^(Th1) cells were determined in BALF together with an array of pulmonary Th1/Th2 cytokines.

**Figure 7:**
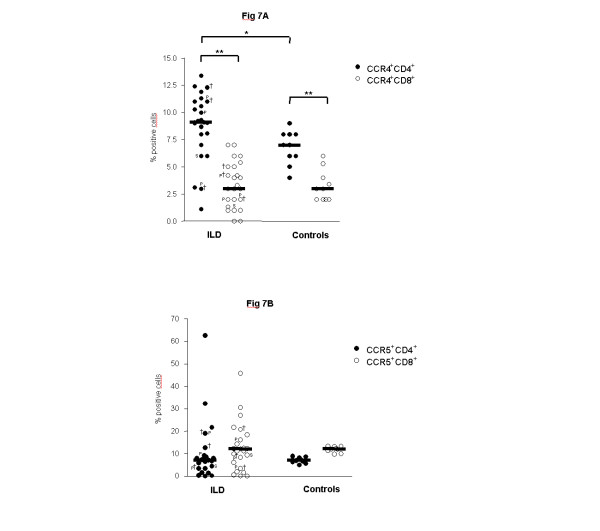
**Pulmonary CCR4^+^, CCR5^+^, and CXCR3^+ ^T cells**. Percentages of (A) CCR4^+^CD4^+^, CCR4^+^CD8^+^, (B) CCR5^+^CD4^+ ^and CCR5^+^CD8^+ ^and (C) CXCR3^+^CD4^+ ^and CXCR3^+^CD8^+ ^T cells in bronchoalveolar lavage fluid (BALF) are shown in children with interstitial lung diseases (ILD) and healthy controls. Percentages of CCR4^+^CD4^+^, CCR4^+^CD8^+^, CCR5^+^CD4^+^, CCR5^+^CD8^+^, CXCR3^+^CD4^+ ^and CXCR3^+^CD8^+ ^T cells were analyzed in BALF by flow cytometry. Median values are shown by horizontal bars. Differences between the patient groups were tested with the Mann-Whitney *U *test; * p < 0.05; ** p < 0.01

Children with ILD had significantly higher percentages of CCR4^+^CD4^+ ^(Th2) cells as compared to control children (Figure 7A). CCR4 was predominantly expressed on CD4^+ ^cells. The majority of CCR4^+^CD4^+ ^cells had an activated phenotype (68% CCR4^+^CD69^+^). CCR3^+ ^(Th2) cells were not detectable in BALF. Percentages of CCR5^+ ^and CXCR3^+ ^T cells (both Th1) were low and did not differ among the patient groups (Figures 7B, C).

Levels of IFN-γ were increased in ILD patients (p < 0.05), whereas the remaining cytokines did not differ among the patient groups (data not shown).

## Discussion

The present work demonstrates that BALF levels of MCP-1 are consistently increased in pediatric ILD. This is accompanied by increased frequencies of the corresponding CCR2 ^+ ^T cells. Levels of MCP-1 and frequencies of CCR2^+ ^T cells were higher in fibrotic than in non-fibrotic forms of ILD and correlated with restrictive lung function parameters and ILD disease severity, indicating a relevance of the MCP-1/CCR2 axis in the pathogenesis of pediatric ILD. Infiltrating T cells are a characteristic feature of pulmonary tissue from ILD patients[45] and T cells in BALF were found to correlate with T cells in pulmonary tissue[46]. In line with previous findings[47,48], T cells were increased in BALF of our children with ILD as compared to control patients, suggesting a contribution of T cells to the pathogenesis of pediatric ILD. Studies in adult patients indicated that MCP-1 plays a role in the pathogenesis of different forms of ILD, including IPF[9,12,13], PAP[11,14], sarcoidosis[12], scleroderma with lung involvement[49] and granulomatous lung diseases[50]. Serum levels of MCP-1 were significantly elevated in adult patients with ILD[9,51] and were closely related to the clinical course[9]. However, as outlined above, ILD in children differs noticeably from ILD in adulthood. Pediatric ILD is extremely rare and little data exist with respect to pathoimmunological mechanisms. Thus, it is very hard to study a large patient group and to find enough children for each ILD subtype. We found elevated levels of MCP-1 and CCR2^+ ^T cells in various etiologies of ILD, which suggests a common pulmonary T cell response for various forms of pediatric ILD.

Thus far, frequencies of BALF CCR2^+ ^T cells in human ILD have not been determined. The parallel increase of MCP-1 and CCR2^+ ^T cells in BALF of ILD children further substantiates the importance of this chemokine and its receptor in the pathogenesis of ILD, as suggested by mouse models. In these models, the relevance of the MCP-1/CCR2 interaction was mainly addressed with respect to pulmonary fibrosis. Our children with pulmonary fibrosis had increased levels of MCP-1 and increased percentages of CCR2^+ ^cells compared to children with non-fibrotic ILD. However, MCP-1 levels and percentages of CCR2^+ ^T cells were elevated both in fibrotic and non-fibrotic ILD children as compared to controls. In addition, MCP-1 and CCR2^+ ^T cells were also elevated in pediatric PAP that usually does not progress to pulmonary fibrosis. In fact, one of the three patients with the highest levels of MCP-1 and CCR2^+ ^T cells had PAP without any indication of fibrosis. Similar observations were made recently for MCP-1 in adult PAP patients[11]. A possible biological relevance of MCP-1 levels and CCR2^+ ^T cells in pediatric ILD is further suggested by their correlation with restrictive lung function parameters and the ILD disease severity score and by the finding that the deceased children with the most severe course of disease exhibited the highest BALF levels of these markers. The possibility that MCP-1 and CCR2^+ ^T cells are a general phenomenon of pediatric lung diseases seems very unlikely, since these markers were present only at low levels in BALF of children with severe allergic asthma. This is in line with findings in an *Aspergillus*-induced allergic mouse model, where a Th2-mediated lung pathology occured in the absence of MCP-1 or CCR2[52].

To assess the value of CCR2^+ ^T cells and MCP-1 levels in the longitudinal course of children with ILD, three consecutive BALs were performed in four children with ILD including three ILD patients who died and one patient who stayed clinically stable. The three deceased children had high and continuously rising levels of MCP-1 and CCR2^+^CD4^+ ^T cells, while the stable patient had low levels of MCP-1 and percentages of CCR2^+^CD4^+ ^T cells. Thus, levels of MCP-1 and percentages of CCR2^+^CD4^+ ^T cells might reflect the disease progression in pediatric ILD.

Interestingly, immunosuppressive treatment was not associated with altered levels of MCP-1 and CCR2^+ ^T cells in BALF (data not shown). This is in contrast to a study of Suga et al.[9] in adult ILD patients where serum levels of MCP-1 were closely related to the effectiveness of corticosteroid therapy. Given the assumption that MCP-1 and CCR2 are important players in the pathophysiology of ILD in children, the lack of association with corticosteroid therapy might explain, at least in part, why corticosteroids are sometimes unable to control the progression of pediatric ILD.

Several studies indicated that MCP-1 and CCR2 are involved in Th1[53,54] and Th2 immunity [55-58]. Furthermore, it has been suggested that ILD and pulmonary fibrosis are associated with a Th2 immune response[20-22,59-61]. Experiments in mice showed that a lack of MCP-1[62] leads to decreased Th1 responses while MCP-1 over-expression[58] results in increased levels of Th2 cytokines. Th1/Th2 cytokine levels in BALF were low or undetectable in BALF of our children. However, CCR4^+^CD4^+ ^T cells were moderately but significantly elevated in ILD patients. On the other hand, CCR4^+^CD4^+ ^T cells are clearly less frequent in ILD compared to allergic asthma[42]. Thus, a strong Th2 response seems unlikely in our ILD patients. Beneath T-cells, MCP-1 attracts CCR2^+ ^monocytes/macrophages[63]. In mouse models, MCP-1 was found to attract monocytes to the inflamed lung, which was accompanied by a concomitant downregulation of pulmonary MCP-1 levels[64]. We found no difference in the percentage of CCR2^+ ^alveolar macrophages in BALF between children with ILD and control children or between fibrotic and non-fibrotic forms of ILD (data not shown). Instead, we found a strong correlation between percentages of CCR2^+ ^T cells and levels of MCP-1 in BALF of ILD patients. Therefore, we assume that pulmonary MCP-1 acts on CCR2^+ ^T cells, which accumulate in the BALF of children with ILD.

## Conclusion

In conclusion, CCR2^+ ^T cells and levels of MCP-1 are characteristic components in BALF of children with ILD. A pathophysiological role in pediatric ILD seems likely as their levels relate to restrictive lung function and ILD disease severity. Therefore, pharmacological targeting of the MCP-1/CCR2 axis might represent an additional option for the treatment of ILD in children.

## Abbreviations

BAL(F): Bronchoalveolar lavage (fluid)

CC: CC chemokine receptor

CXC: CXC chemokine receptor

FVC: Forced vital capacity

IFN-γ: Interferon-γ

IL-: Interleukin

IPF: Idiopathic pulmonary fibrosis

IPH: Idiopathic pulmonary hemosiderosis

LIP: Lymphocytic interstitial pneumonia

MCP-1: Monocyte chemotactic protein 1 (CCL2)

PAP: Pulmonary alveolar proteinosis

TGF-β: Transforming growth factor β

Th1/Th2: T helper cell 1/2

TLC: Total lung capacity

TNF-α: Tumor necrosis factor-α

## Competing interests

The author(s) declare that they have no competing interests.

## Authors' contributions

DH carried out the experimental analyses and wrote the manuscript. MG characterized the study population, performed bronchoalveolar lavage and participated in the study design. TN performed bronchoalveolar lavage and patient characterization. GZ and CP participated in the experimental analyses. DR and DJS participated in the study design and reviewed the manuscript. SKE designed the study, supervised the experimental analyses and wrote the manuscript. All authors read and approved the final manuscript.
